# Benefits and challenges of incorporating citizen science into university education

**DOI:** 10.1371/journal.pone.0186285

**Published:** 2017-11-01

**Authors:** Nicola Mitchell, Maggie Triska, Andrea Liberatore, Linden Ashcroft, Richard Weatherill, Nancy Longnecker

**Affiliations:** 1 School of Biological Sciences, The University of Western Australia, Crawley, WA, Australia; 2 Centre for Science Communication, University of Otago, Dunedin, New Zealand; 3 Earthwatch Institute, Melbourne, Australia; Utrecht University, NETHERLANDS

## Abstract

A common feature of many citizen science projects is the collection of data by unpaid contributors with the expectation that the data will be used in research. Here we report a teaching strategy that combined citizen science with inquiry-based learning to offer first year university students an authentic research experience. A six-year partnership with the Australian phenology citizen science program *ClimateWatch* has enabled biology students from the University of Western Australia to contribute phenological data on plants and animals, and to conduct the first research on unvalidated species datasets contributed by public and university participants. Students wrote scientific articles on their findings, peer-reviewed each other’s work and the best articles were published online in a student journal. Surveys of more than 1500 students showed that their environmental engagement increased significantly after participating in data collection and data analysis. However, only 31% of students agreed with the statement that “data collected by citizen scientists are reliable” at the end of the project, whereas the rate of agreement was initially 79%. This change in perception was likely due to students discovering erroneous records when they mapped data points and analysed submitted photographs. A positive consequence was that students subsequently reported being more careful to avoid errors in their own data collection, and making greater efforts to contribute records that were useful for future scientific research. Evaluation of our project has shown that by embedding a research process within citizen science participation, university students are given cause to improve their contributions to environmental datasets. If true for citizen scientists in general, enabling participants as well as scientists to analyse data could enhance data quality, and so address a key constraint of broad-scale citizen science programs.

## Introduction

Active engagement of undergraduate science students is essential for increasing students’ abilities to connect concepts such as environmental issues to everyday life and for increasing their environmental knowledge [1, 2]. Strategies such as inquiry-based learning, which promotes the retention of material by increasing deep thinking in students, may be used to accomplish this [3, 4]. Citizen science offers the potential to increase student engagement through active and inquiry-based learning; consequently, citizen science programs have been implemented into some undergraduate classes and research [5–7].

One frequent aim of citizen science programs is to increase science and environmental literacy [8–11]. Many citizen science programs directly involve everyday people—usually volunteers—in the collection of data that can be used productively by professional scientists [10, 12–17]. Less often, citizen science involves co-production of knowledge [14] in which citizens and professional scientists are partners in various stages of the scientific process, from setting research questions to analysis and discussion of results [18, 19]. Mutual benefits to citizen science programs and universities can be achieved by involving university students in data collection and analysis. Indeed, a lack of participation by young people in citizen science programs has led to calls to recruit citizen scientists from the university education sector [20].

Assignments incorporating the Australian citizen science program *ClimateWatch* (http://www.climatewatch.org.au) were introduced to first year biology units in 2011 at The University of Western Australia (UWA). *ClimateWatch* is a phenology program developed by Earthwatch Australia that invites participants to monitor the timing of seasonal events in 185 species of plants, animals, fungi and algae, to track potential changes in life cycles and/or distributions as the climate changes. These species are usually conspicuous and are known as ‘indicator’ species, and represent a small portion of the biodiversity in any given area of Australia. Sightings can be made anywhere, or at predetermined *ClimateWatch* walking trails. Since its launch in 2009 *ClimateWatch* has engaged over 20,000 participants who have submitted more than 95,000 observations, with a large proportion of participants being university students enrolled at Australian universities.

**Fig 1:**
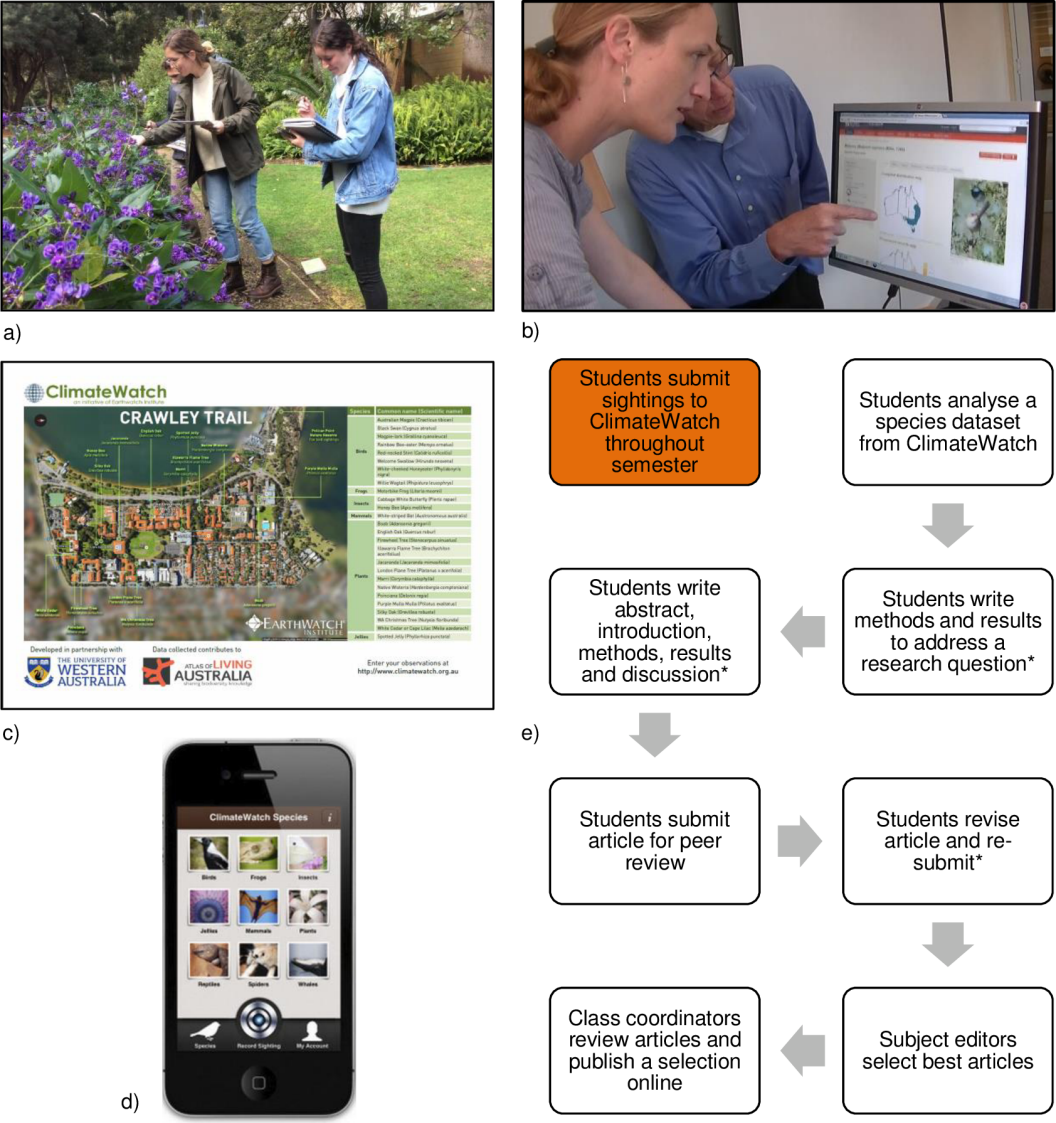
(a) Students on a ClimateWatch trail on the Crawley campus of the University of Western Australia; (b) students conducting research for the Journal Project; (c) the UWA Crawley Trail map where indicator species are introduced to students; (d) the submission screen on ClimateWatch’s smartphone app; and (e) a summary schema of ClimateWatch activities and the Journal Project.The orange box indicates the responsibility for collection of phenological data on local plants and animals, which has occurred since 2011, and white boxes show the components of the Journal Project, which were added from 2012. Asterisks indicate where feedback was given from subject editors (formative assessment) and where mark components were awarded.

First year (freshmen) students enrolled in a biology class in 2011 were required to submit records of *ClimateWatch* indicator species to receive a small proportion of a class grade. From 2012 to 2014 a similar assignment was given to students enrolled in a larger biology class, but students were further required to analyse all citizen science data on a particular indicator species submitted to *ClimateWatch* since the program’s inception, and did this research in small teams. Teams wrote a journal article focusing on the species’ potential response to climate change, the validation of citizen science datasets, or a combination of both topics. This semester-long assignment, hereafter known as the ‘Journal Project’ (Fig 1), included a requirement to compare the citizen science data with distributional and phenological data located in peer-reviewed literature and online databases, such as the Atlas of Living Australia (ww.ala.org.au). The Journal Project design follows Berkowitz et al. [2] who suggested that pedagogy strategies such as these can increase students’ environmental knowledge.

Student attitudes and learning were assessed via two surveys: one prior to their participation (pre) and one at the end of the semester (post). The surveys asked students about their knowledge and exposure to their local environment, their previous involvement in citizen science projects and about their perceptions of whether they achieved the learning outcomes of the *ClimateWatch* assignment(s). We expected that student participation in citizen science and the addition of the Journal Project would result in students being able to: 1) engage with their environment, 2) recognise and record phenological information on indicator species occurring in Western Australia, 3) gain experience with process for publication of scientific findings, and 4) improve their understanding of climate change and its potential effects on biota. In this paper, we reflect on whether these educational objectives were achieved, and recommend ways in which the quality of data submitted by students to citizen science programmes can be improved.

## Materials and methods

### Introducing students to citizen science

Students were introduced to *ClimateWatch* in Week 2 of the semester via a self-guided walking trail–either on UWA’s Crawley Campus in Perth, or near a small regional campus in Albany, where about ten students enrol each year. These trails are publicly accessible (http://www.climatewatch.org.au/trails/uwa-crawley; http://www.climatewatch.org.au/trails/lake-seppings), and include many indicator species–primarily plants. Students were issued with field guides on the species they were likely to encounter on each trail to assist with identification. While walking the trails, most students trialled a smartphone application for submitting sightings (available from 2012), and once back in class were shown how to submit sightings using the *ClimateWatch* website interface. Following this introduction, students were encouraged to submit sightings of species in their neighbourhoods across the expansive Perth metropolitan area [21], on other *ClimateWatch* trails, in regional parks and reserves, and more generally in any travel throughout Australia. From 2012 they were discouraged from recording further observations on the Crawley and Lake Seppings trails, to avoid clusters of data in time and space. Students were required to independently submit a set of sightings (Table 1), to *ClimateWatch* by the end of the 13-week semester and usually received a small portion of their unit mark for completing this task.

**Table 1:** Details of citizen science assignments in UWA biology classes from 2011–2014, highlighting points of difference between years and showing the number of students surveyed. Assignment design in 2015 and 2016 was similar to 2014, but students were not surveyed in these years.

Year		2011	2012	2013	2014
Class code		BIOL1131	BIOL1130	BIOL1130	BIOL1130
Number of enrolled students		266	484	577	586
Available data input mechanisms*	Website	Yes	Yes	Yes	Yes
	iPhone app	No	Yes	Yes	Yes
	Android app	No	No	Yes	Yes
Assessment task (proportion of final grade, %)		20 sightings (5)	20 sightings (5)	10 sightings, all of different species (0, but non-compliance reduced other marks)	10 sightings, all of different species, or a species in two phenophases. Data reliability assessed by demonstrator (5)
Journal Project? (proportion of final grade, %)		No	Yes (20)	Yes (30)	Yes (40)
Number of student responses to pre-survey		141	430	465	395
Number of student responses to post-survey		218	364	332	485

* The addition of *ClimateWatch* smart phone applications allowed data to be submitted in real time with accurate GPS data, and it became easier to submit images with each sighting.

### The Journal Project: Expanding inquiry via student evaluation of citizen science data

From 2012, students were divided into teams early in the semester and provided with a raw dataset on a *ClimateWatch* indicator species (usually a plant or an animal). Each dataset consisted of all records of that species submitted to *ClimateWatch* since the launch of its website in September 2009, and had not been subject to any quality control. Many of the observations available for analysis in 2012 were collected by the 2011 UWA students; while from 2013 onwards the datasets have had a more national coverage and have increasingly included sightings submitted by university students at other Australian universities, from public citizen scientists, as well as from previous cohorts of UWA students.

Teams were encouraged to develop methods to detect any unreliable records in a dataset, and to think about questions that could be tested with each dataset. Teams were prompted to consider two question themes that they could usefully address: 1) does their data provide evidence of a phenological or distributional shift when compared to historical records (defined as records collected prior to the initiation of *ClimateWatch*), or 2) does citizen science produce reliable data? A draft research article was submitted by the team midway through semester, and these articles were distributed for peer review, where all students in the class were asked to produce a constructive review of another team’s article. Peer reviews were assessed, and provided a means for self-reflection. Peer reviews were returned to the team along with a more substantive review by a ‘Subject Editor’—a PhD student or postdoctoral fellow who had publication experience, was familiar with the species dataset and who assessed all the submissions on that particular species. Each team’s article was revised based on the feedback they received, and was submitted in Week 12. The best articles on each species dataset were selected by the editors and class coordinators and published in an online student journal *Cygnus* on the final day of semester (end of Week 13). Further information on the journal and 130 student articles published between 2012 and 2016 can be accessed online [22].

### Surveys of student engagement and learning

Surveys of students were conducted from 2011–2014; a pre-survey was conducted before their introduction to *ClimateWatch*, and a post-survey was conducted in the final week of semester. Both surveys were administered online using SurveyGizmo (http://www.surveygizmo.com/). Student responses were de-identified, as respondents provided only the last four digits of an eight digit student number for data matching purposes. In the pre-survey, students were asked to: 1) provide basic personal information such as their age, place of birth, and study path; 2) outline any previous exposure to citizen science; and 3), to evaluate a range of statements on citizen science. The post-survey was more extensive, and included additional questions seeking feedback on their experiences with *ClimateWatch*, and from 2012 also included statements that evaluated the learning objectives of the Journal Project. Three statements were common to the pre- and post-surveys, termed ‘paired statements’. Quantitative responses included either a forced choice Likert scale (Strongly disagree, Disagree, Agree, Strongly agree) or yes/no options (Table 2). For quantitative analysis, Likert scale responses were grouped into Disagree (Strongly disagree and Disagree) and Agree (Strongly agree and Agree). A range of open-ended questions were also asked, to collect qualitative data.

**Table 2:** Statements evaluated by students, grouped by theme. All statements were evaluated on a Likert scale unless indicated otherwise.

Theme	Survey Statement	Paired?
***Environmental engagement***	• After participation with the *ClimateWatch* program, I am more engaged with the environment	
	• Writing a peer-reviewed journal article, using *ClimateWatch* data, has expanded my understanding of species and their potential responses to climate change	
	• Producing a peer-reviewed journal article increased my interest in publishing biological research	
***Behavior as a citizen scientist***	• Did analysing *ClimateWatch* data affect your approach to data collection? (yes/no)	
	• Collecting and entering data for *ClimateWatch* made me more aware of species presence and behavior	
	• I plan to continue to participate in *ClimateWatch*	
	• I have introduced others to *ClimateWatch* (yes/no)	
	• I plan to introduce others to *ClimateWatch*	
	• I plan to participate in other citizen science programs	
***Training in scientific practice***	• I'm interested in reading peer-reviewed journal articles	Yes (3)
	• Feedback from a peer was helpful in preparing my team's article	
	• I found it useful to work in small teams for each phase of the Journal Project	
	• Feedback from a subject editor was helpful in preparing my team's article	
***Citizen science importance and reliability***	• Data collected by citizen scientists are reliable*	
	• Data collected by citizen scientists are used by professional scientists *	Yes (1)
	• It is useful to involve citizen scientists in scientific research*	Yes (2)
	• The Journal Project identified potential challenges with large scale citizen science data collection	
	• The Journal Project identified potential opportunities provided from large scale citizen science data collection	

**Fig 2:**
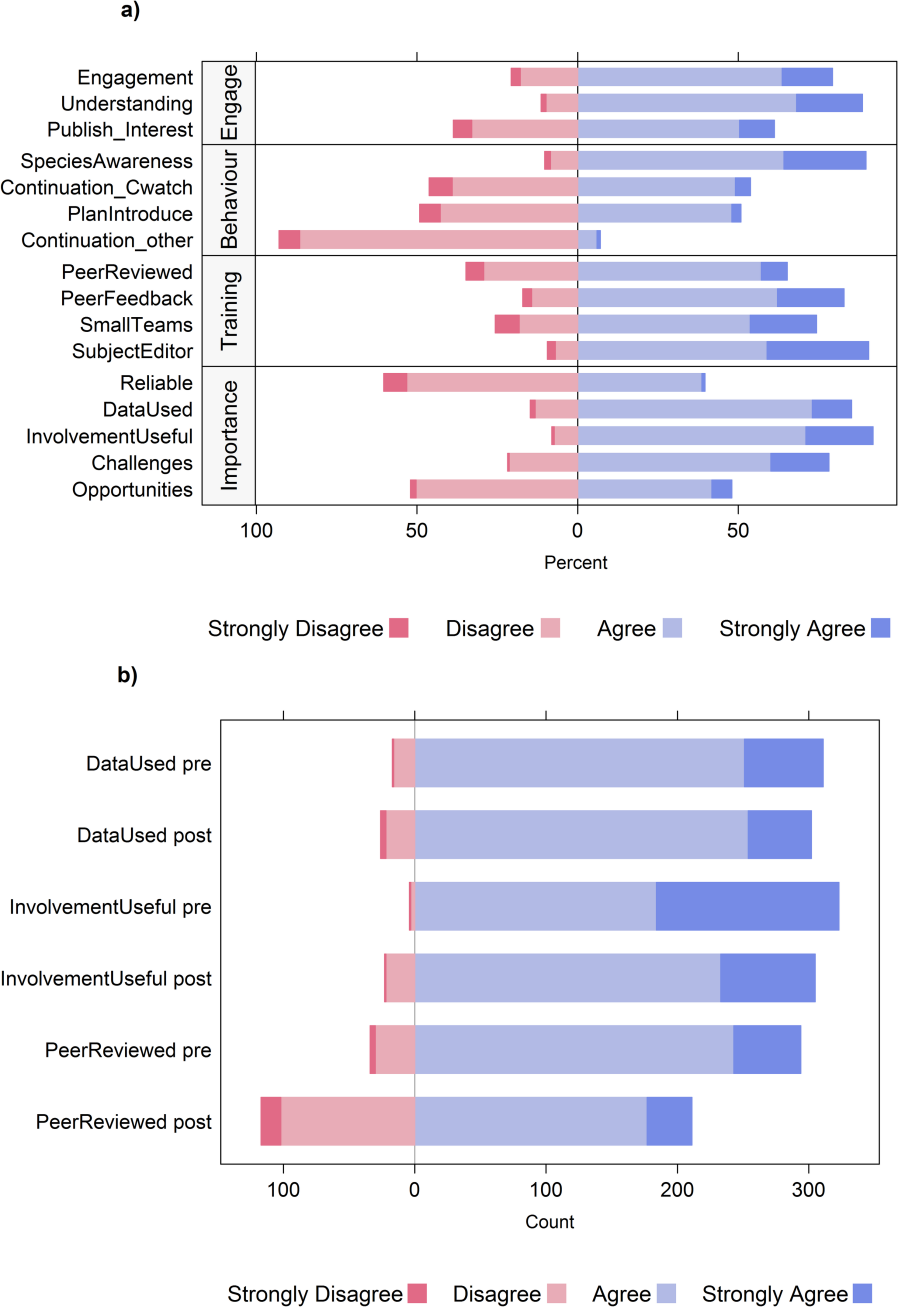
Student evaluations of the statements listed in Table 2. a) Relative agreement with the Likert-scaled statements listed in Table 2, completed after both the *ClimateWatch* and Journal Project assignments; b) paired comparison between the pre and post surveys of agreement on three statements (refer Table 2), showing some shift in student responses over the semester. All data shown in panel a) are pooled from the 2012–2014 students.

Students enrolled in 2012–2014 were asked to evaluate all statements, whereas the 2011 students only evaluated statement indicated with an asterisk*, as they did not experience the Journal Project. A paired statement was evaluated in both the pre- and post-surveys, and numbers refer to the order the data are presented in Fig 2.

Responses to pre and post surveys were matched via the partial student number, and any duplicate responses (identified by identical numbers), were removed, which resulted in 5, 10, 3, and 11% of surveys being discarded in 2011, 2012, 2013 and 2014, respectively. If one of the duplicates was a partially completed survey, it was removed preferentially. Otherwise, if duplicate surveys were similar, then one was removed at random. When evaluating paired statements, any survey without a match was discarded (51, 10, 17 and 32% of surveys in 2011, 2012, 2013 and 2014, respectively), leaving 1038 paired responses for comparison. The use of these survey data for research was judged to be exempt from ethics review under Australian and university policy, as it was based on an existing collection of data that only contained non-identifiable data about human beings (ref RA/4/1/9130: Human Research Ethics Office, The University of Western Australia). The students pictured in this manuscript have given written informed consent (as outlined in PLOS consent form) to publish their images.

Given that the 2011 students did not experience the Journal Project, we restricted our analysis of student responses to the statements evaluated by the 2012, 2013 and 2014 students. No trends based on study year were detected, and hence the 2012–2014 survey data were subsequently pooled for analyses. Next, the cleaned data were grouped into four categories of statements (*Environmental engagement*, *Behavior as a citizen scientist*, *Training in scientific practice*, and *Citizen Science importance and reliability*, Table 2) and the percentage of each forced Likert scale response was calculated. Statements evaluated with a forced Likert scale were plotted using package “HH” [23] in the open-source software program R [24].

To determine if student responses to paired statements were different after the *ClimateWatch* assignment and Journal Project, we used a Wilcoxon Signed-Rank Test, which is a nonparametric test that compares the median values of pairs to determine if they are significantly different. This test assumes that the data were paired and collected at different survey times and is similar to a paired *t*-test in parametric statistics. Wilcoxon Signed-rank tests were completed using base R and package “Coin” [25] to determine the effect size of the difference in the medians between survey times.

Content analysis was performed on student responses from six open-ended questions asked in the post survey. Thematic coding was used to isolate themes within the responses, and a coding manual was developed for each question. Each coding manual was validated by testing a randomly-selected subset of 20 responses between two authors (AL and NL; S1 Table). Intercoder reliability was calculated with percentage agreement and Krippendorff’s alpha [26] which provides a statistical measure of reliability by comparing reproducibility of results from different coders. Coding manuals were revised and tested again until a minimum of 90% agreement and 0.74 for Krippendorf’s alpha was reached for each theme. Once coding manuals for each survey question were validated, all student responses were coded and analysed.

## Results and discussion

University students engaged in *ClimateWatch* assignments have made major contributions to the phenological data available for Australian species. For example, by November 2013, 45% of all *ClimateWatch* records came from UWA students, with an additional 22% of records submitted by students from eight other Australian universities, and 33% of records submitted by the ‘public’ (i.e. citizen scientists who were not associated with university-based assignments). This breakdown was similar by November 2014; UWA students contributed 41% of records, 27% of records were submitted by students from other universities, and 33% of records came from the public. Hence around two-thirds of *ClimateWatch’s* records between 2011 and 2014 were submitted by students enrolled in universities using *ClimateWatch* as part of a teaching program, while the remaining one-third were records from a broader community of citizen scientists. The typical student in a UWA biology class was female, was aged <20 years, grew up in a suburban environment and was born in Western Australia, and there was little variation in this pattern between years. Between 16 and 26% of the student cohort in any one year was born overseas (see S2 Table for demographic data).

### Environmental engagement

We inferred that students enjoyed participating in *ClimateWatch* because the majority (55%) planned to continue to record observations and more than 35% had introduced others to *ClimateWatch* (Fig 2A). These findings indicate that students were interested in the assignment and the collection of citizen science data beyond the classroom, and around 80% agreed that participation in the *ClimateWatch* program increased their environmental engagement. One student noted, “It forced me to go outside and observe the environment which turned out to be a really lovely thing to do.” This supports other research that participation of individuals in citizen science can increase awareness of biological issues, and encourages their participation in further research projects [18, 19, 27]. Reported advantages of collecting *ClimateWatch* data (Table 3) included learning about species and the local environment (56%), contributing to science (25%), spending more time outdoors (3%) and gaining an introduction to data collection or citizen science (3%). Students were also able to articulate a suite of opportunities that arise out of citizen involvement in data collection such as the data being useful to science and scientists (65%), that the data could potentially increase our ability to detect effects of climate change (25%) and that there are benefits to society including interaction with the environment, awareness of climate change and work experience for those pursuing scientific careers (12%).

**Table 3:** Themes, examples and percentages of responses falling into each theme for open ended questions asked of students from 2012–2014.

Theme	% Response	Example
What were the main advantages of collecting or entering data for *ClimateWatch*? (n = 1180)		
Learning	58	I was amazed by the variety of species, particularly plants and birds, that live so close to my home that I previously have hardly noticed
Contributing	25	I feel like I am taking part in important environmental research
Easy	12	It was easy to do when I went for walks
Environment	3	Experiencing nature when I otherwise wouldn't have
Introduction	3	It allowed me to explore the idea of becoming a field scientist by allowing me to conduct research
Other	6	Allowed practical application of my biology skills
The Journal Project identified potential opportunities provided from large scale citizen science data collection. What opportunities did you identify? (n = 572)		
Useful to science	65	It provides a cheap way to collect a lot of information
Detect change	25	Over time, data would show patterns relevant to scientists in identifying links to climate change
Useful to society	12	Opportunities for people to connect with the environment
Other	6	The opportunity to find ways of making citizen scientist data more reliable
The Journal Project identified potential challenges with large-scale citizen science data collection. What challenges did you identify? (n = 923)		
Reliability	85	It isn't always reliable. People record the wrong place or the wrong species accidentally
Data volume	18	The data set is not currently large enough to identify any useful trends
Falsification	4	Faulty sightings due to compulsory nature of students’ assignment (unwilling but must complete sightings)
Other	7	Finding areas to look for native species
Did analysing *ClimateWatch* data affect your approach to data collection for our 20 observations? (Yes) How did you change your approach to the data collection and reporting? (n = 922)		
Increase usefulness	89	More meticulous in the collection of data. The devil is in the details
Data analysis	5	It made me look for more sources to back up or reject the data that were collected
Other	8	I realised that it's important to attach a photograph so the sighting can be verified
Did analysing *ClimateWatch* data affect your approach to data collection for our 20 observations? (No) Why not? (n = 273)		
No change	39	[ClimateWatch] did not really contain any information about how best to go about collecting data. . . my approach was as good as any citizen scientist's
Logistics	21	Most of my sightings were recorded before the article was written
Already good	15	Because I had already decided to take photos and be sure that I was accurate before I started
No point	8	The data I received for the cabbage white [a butterfly] was so skewed. . . made me wonder what the point is of me entering 'accurate' results
Other	17	Because I had to do it

Citizen scientists often report that they improve their scientific knowledge and literacy via their participation [19]. In this study, over 85% of students reported that they were more aware of at least one species and its behavior since being exposed to *ClimaetWatch* (Fig 2A). As one student said of their participation, it “creat[ed] awareness of the different kinds of plants and birds. They are not just trees, they are now jacarandas and banksia and birds are not black-tail bird or crows they are willie wagtails and magpie lark etc.” Students reported becoming more aware of their local environment, writing “you learn that there is life everywhere you look” and “[I gained] knowledge of how fragile the environment is.” As *ClimateWatch* requires participants to collect data on relatively common species, they are not only able to identify the species, but become more aware of the species’ phenology (i.e. nesting, breeding, flowering, and migration). Therefore the completion of the data collection, the analysis involved in writing a journal article, and the process of peer reviewing another article improved students’ understanding of potential species’ responses to climate change, as 89% of students reported in the survey in 2012. For example one student said, “[I] became aware of those species that will be affected by climate change–[the assignment] puts climate change in perspective”. Hence, although the Journal Project was challenging for a first year university student, and had the potential to decrease students’ interest in environmental issues, its net effects were positive, with most students (61%) indicating that producing a peer-reviewed journal article had increased their interest in publishing biological research.

### Behavior and training in scientific practice

The assignment served as an introduction to citizen science; “[it introduced] us to citizen science and opened the door for future opportunity as citizen scientists”. Many students also enjoyed having the opportunity to contribute to science as part of an assignment, saying “knowing that these data will be used by scientists for research makes me feel useful” and “I feel I’m making a small impact on saving animals and plants from the ill effects of climate change. This is very rewarding.” One student noted that they “felt involved in the learning process.” Moreover the assignment provided an authentic research experience, with students saying that they gained “hands-on experience [with] collecting data”. A majority (55%) of students indicated their intention to continue to contribute to ClimateWatch while most (90%) disagreed that they would continue to contribute to another citizen science program. It is possible that this might change if students were made aware of other opportunities. Other studies of citizen science have found that participants often continue their involvement in citizen science programs [2, 5, 28, 29], indicating the likelihood of acting on some of the stated intentions of participants in this study to continue contributing to citizen science.

The majority of students (74%), found it useful to work in small teams for each phase of the Journal Project, and thought that their scientific articles were improved by feedback from a subject editor (91%) and their peers (83%). In the pre-survey most students (86%) were interested in reading peer-reviewed journal articles but there was diminished interest (–20%) by post-survey (comparison of Likert scale values; W = 19960.00, Z = -11.4946, p<0.01, r = 0.38; respectively, r = effect size, see Fig 2B). In the absence of other evidence, we suggest that decreased reported interest in reading scientific articles at the end of the semester could be related to heavy study loads at this time. Alternatively, some students could have lost confidence in their ability to comprehend scientific articles after their immersion in the Journal Project. At UWA, these skills are taught in core first year units that all science undergraduate students take, but we could consider incorporating a specific pedagogical strategy to improve engagement with primary literature, such as the C.R.E.A.T.E. method developed for biology students at the City College of New York [30].

### The value and reliability of citizen science data

When first surveyed, most students felt it was useful to involve citizen scientists in scientific research (92%), and broadly agreed that data collected by citizen scientists are used by professional scientists (95%). After completing the Journal Project, there was a decreased belief that citizen science data are used by professional scientists and that citizen scientists are useful in scientific research (Fig 2B). In both cases the Likert scale values in the post survey were significantly lower than the initial Likert scale values (–8.1%, W = 15069.00, Z = –6.92, p<0.01, r = 0.23; –6.9%, W = 14905.50, Z = –11.11, p<0.01, r = 0.38; respectively). Exposure to citizen science and the experiential learning provided by the Journal Project caused most students (78%) to identify challenges with large-scale data collection by citizen scientists, but 48% of students also thought there were opportunities to be gained from citizen science data. The fact that some datasets were not necessarily large enough to answer focal research questions would have contributed to conflicting perceptions about whether citizen science is a useful exercise, an issue that will resolve for *ClimateWatch* over time with continuing contributions. Further, as *ClimateWatch* is a relatively new citizen science program without a track record of peer-reviewed publications in established journals, students had limited insight into how the datasets could be used in research.

When asked an open-ended question about the challenges of citizen science data collection, 85% of students questioned the data’s reliability, with an additional low but concerning 4% suggesting that mandatory submission of data by students might lead to falsification (Table 3). In the post-surveys, fewer than half of the 2012–2014 students (40%) reported that data collected by citizen scientists were reliable (Fig 2A), noting in their open-ended responses that “people record the wrong place or the wrong species accidentally” and “we cannot be sure how educated citizen scientist are, and therefore uncertainty [of data] is much higher”.

Most students (77%) agreed that their approach to data collection changed after analysing *ClimateWatch* data. Of these students, 86% reported that they strove to supply more accurate and useful data. Of the 23% of students who did not change their approach, 54% believed that their approach to data collection was already good enough. A notable finding is that the 2011 students had much greater confidence in the reliability of data collected by citizen scientists. A large proportion (75%) of these students agreed or strongly agreed with the statement that data collected by citizen scientists is reliable in the post-survey. This compared to 41% of students in 2012 (χ^2^ = 30.27, df = 1, p<0.01), 33% in 2013 (χ^2^ = 46.54, df = 1, p<0.01) and 43% in 2014 (χ^2^ = 29.08, df = 1, p<0.01; data aggregated in Fig 2A). The key difference here is that the 2012–2014 students analysed and wrote a scientific article based on data collected by other citizen scientists, which were deliberately provided by *ClimateWatch* in an invalidated form to allow students to assess data reliability. Errors became more apparent in later years due to more records, including images submitted from mobile phones (discussed in articles published in *Cygnus* from 2014 [22]). The lower confidence of the 2012–2014 students possibly also reflected frustration that some datasets provided for analysis showed little variation. This was especially true in 2012, as these students were primarily analysing data submitted by the 2011 UWA students, which consisted of 5566 sightings, about half of which were of common birds or trees occurring on the Crawley *ClimateWatch* trail.

Encouragingly, perception of poor quality data by students was positively correlated with a student’s likelihood to continue participating in citizen science programs (*T* = 0.31, *p* = <0.01, R^2^ = 0.09), suggesting that students with the greatest awareness of the limitations of poor quality data were more likely to continue to contribute data themselves. Furthermore, approximately 75% of students reported that the process of scrutinising a species’ dataset (via species distribution mapping, and/or analysis of submitted images; see student articles in [22]) had influenced their own approach to data collection. When asked *how* analysis of a dataset changed their own data collection, most students indicated they collected more accurate data, and tried to make it more meaningful for phenological analysis (e.g. they would try to record a bird breeding rather than a bird feeding). Students said “[the assignment] made me more aware of what constitutes useful data and what doesn’t” and “[it] made me more critical of what I thought I was seeing so I could produce higher quality sightings.” It is possible that students to some degree self-report improving accuracy because it is what they think they were meant to say. Nonetheless self-reported behavior can be a useful indicator of actual behavior in many situations [29, 31]. This study’s self-reported data indicate at the least an awareness of the importance of accuracy and that there is potentially an effect on actual behavior. Comparing self-reported improvement in accuracy with measured accuracy would be a fruitful focus of further research.

Given that more than 95,000 sightings have been submitted to ClimateWatch (as of November 2016), we are not able to contrast here how the quality of data submitted by UWA students differs to that of other groups of interest (e.g. participants from other universities, public submissions), nor did we map the demographic profile of students on to the patterns we identified in qualitative and quantitative data. This could also be a focus for future analysis, to build on a very small literature on the quality of data submitted by student researchers to citizen science programs [32,33].

Based on our experience of embedding citizen science in teaching, we recommend careful review of student contributions to online datasets–a process we implemented from 2014 (Table 1). Students now present the data they submitted to *ClimateWatch* to their classmates and demonstrator in order to receive unit marks. This process engages the class, encourages students to seek more unusual or more challenging observations, and allows errors to be detected and corrected before inclusion in the dataset. Incorporating feedback capability in data-capture software, such as the use of ‘smart filters’ that flag erroneous records [34] will also lead to improvements in data quality over time. The *ClimateWatch* website and smartphone applications lack this capacity at present, instead relying on identification guides and the selection of easily identifiable species to reduce the likelihood of misidentifications. Nevertheless, tertiary biology students in Australia appear to struggle to identify some indicator species (refer to [22] for examples), even though most students are identifying species from their country and environment of origin (Table 3). Potentially, the high biodiversity in Australia (southwestern Australia, the location of the UWA students, is a biodiversity hotspot; [35]) is one challenge for effectiveness of multi-species citizen science projects that use non-expert contributors. Conversely, it is also a benefit of citizen science programs for which education of participants is an important objective.

There is no doubting the potential value of citizen science data. Dickinson et al. [36] estimated that the value of contributing efforts to one project alone (Cornell Lab of Ornithology’s FeederWatch) was roughly worth the equivalent of $3,000,000 per year. But is the quality sufficient to warrant its use in scientific studies? The answer would appear to be yes. Data collected by citizen scientists in eight large programs have contributed to about 1110 peer-reviewed publications and technical reports and PhD theses [36]. A recent meta-analysis detected almost 2000 publications based on citizen science datasets [17]. High quality data are therefore of paramount importance [34], but validation of data is resource-intensive and potentially decreases the benefits of accumulating data at wide spatial and temporal scales. While poor-quality data are less likely to arise in citizen science programs that engage self-motivated volunteers than in projects such as ours where data submission was mandatory, we suggest that increasing the environmental and biological literacy of participants [8, 19] should be a focus of professional scientists who wish to draw upon citizen science for their research. Our study suggests that engaging citizen scientists in the assessment of data quality, and in analysing the data, can be an effective and efficient method of not only validating datasets, but also improving the scientific literacy of citizen scientists, thereby improving the quality of future data they submit.

## Conclusions

In this study we successfully combined citizen science data collection with inquiry-based learning to provide a means to increase environmental engagement of undergraduate students (specifically first year students) and to broaden their environmental and scientific knowledge. Students further learned about the process of data analysis, presentation and publication through the Journal Project, and the 130 articles by student authors published so far [22] are perhaps better testament to the learning outcomes achieved than the analyses presented here. *ClimateWatch* and the Journal Project remain a core part of the first year biology curriculum at UWA, and will continue to be refined and improved. Ideally, we aim to produce new cohorts of citizen scientists who value and record accurate observations for important initiatives such as *ClimateWatch*, and to inspire researchers to work closely with citizen scientists to improve data quality, and in so doing enhance the future impact of their collaborations.

## Supporting information

S1 Table Intercoder reliability assessment for qualitative analysis, showing the percent agreement and Krippendorf’s alpha values for each theme. (DOCX)

S2 Table Demographics (frequency in percentage) of students who completed pre and post surveys in each year of the study. (DOCX)

S1 Data Microsoft Excel database of responses to quantitative survey questions (Likert scale, yes/no) from 2011–2014. (XLSX)

S2 Data Microsoft Excel database of qualitative responses to survey questions from 2011–2014. (XLSX)
