# Supplementary material for: Benefits and challenges of incorporating citizen science into university education
Source: PLoS One. 2017 Nov 1;12(11):e0186285. doi: 10.1371/journal.pone.0186285 (PMC5665417; doi:10.1371/journal.pone.0186285)
Supplement: S1 Table — (DOCX) [file pone.0186285.s001.docx]

**S1 Table** Demographics (frequency in percentage) of students who completed pre and post surveys in each year of the study.

| Student demographics | | Year | | | |
| --- | --- | --- | --- | --- | --- |
|  |  | 2011 | 2012 | 2013 | 2014 |
| (n) | | (107) | (327) | (275) | (328) |
| Gender | Female | 59 | 68 | 69 | 66 |
|  | Male | 41 | 32 | 31 | 34 |
| Age | <20 | 82 | 81 | 81 | 75 |
|  | 21-23 | 9 | 11 | 8 | 14 |
|  | 24-26 | 5 | 2 | 3 | 5 |
|  | 27-29 | 1 | 2 | 3 | 2 |
|  | 30+ | 3 | 4 | 4 | 5 |
|  |  |  |  |  |  |
| Birth place | Western Australia | 79 | 73 | 77 | 69 |
|  | Other Australian state | 3 | 3 | 7 | 4 |
|  | Other country | 19 | 23 | 16 | 26 |
| Environment during childhood | Suburban | 59 | 63 | 65 | 62 |
|  | Inner city | 11 | 11 | 9 | 16 |
|  | Rural | 22 | 15 | 13 | 13 |
|  | Regional | 5 | 8 | 10 | 8 |
|  | Remote | 3 | 1 | 2 | 1 |
|  | Other | 0 | 1 | 1 | 1 |
