# Supplementary material for: Benefits and challenges of incorporating citizen science into university education
Source: PLoS One. 2017 Nov 1;12(11):e0186285. doi: 10.1371/journal.pone.0186285 (PMC5665417; doi:10.1371/journal.pone.0186285)
Supplement: S2 Table — (DOCX) [file pone.0186285.s002.docx]

**S2 Table** Intercoder reliability assessment for qualitative analysis.

|  | Theme | Percent agreement | Krippendorf’s alpha |
| --- | --- | --- | --- |
| What were the main advantages of collecting or entering data for ClimateWatch? (N=1180) | Learning | 100 | 0.91 |
|  | Contributing | 95 | 0.83 |
|  | Easy | 95 | 1 |
|  | Environment | 100 | 0.83 |
|  | Introduction | 100 | 1 |
|  | Other | 95 | 1 |
| The Journal Project identified potential opportunities provided from large sale citizen science data collection. What opportunities did you identify? (n=572) | Useful to science | 100 | 1 |
|  | Detect change | 96 | 0.87 |
|  | Useful to society | 100 | 1 |
|  | Other | 100 | 1 |
| The Journal Project identified potential challenges with large-scale citizen science data collection. What challenges did you identify? (n=923) | Reliability | 95 | 0.88 |
|  | Data volume | 90 | 0.74 |
|  | Falsification | 100 | 1 |
|  | Other | 95 | 0.78 |
| Did analysing ClimateWatch data affect your approach to data collection for our 20 observations? (Yes) How did you change your approach to the data collection and reporting? (n=922) | Increase usefulness | 100 | 1 |
|  | Data analysis | 100 | 1 |
|  | Other | 90 | 0.74 |
| Did analysing ClimateWatch data affect your approach to data collection for our 20 observations? (No) Why not? (n=273) | No change | 95 | 0.9 |
|  | Logistics | 90 | 0.8 |
|  | Already good | 100 | 1 |
|  | No point | 100 | 1 |
|  | Other | 90 | 0.8 |
